# Assessing urban family physician program challenges in Iran: the insurance organizations’ perspective(2021)

**DOI:** 10.1186/s12889-024-19434-5

**Published:** 2024-07-20

**Authors:** Lida Shams, Fatemeh Mohammadi

**Affiliations:** Department of Health Policy and Management, School of Public Health and Safety, Shahid Beheshti University of Medical Sciences, Tehran, Iran

**Keywords:** Health system, Urban family physician, Insurance

## Abstract

**Background:**

The Family Physician Programme is a key health reform in Iran that faces significant challenges in urban areas, particularly in Mazandaran and Fars provinces The study aims to critically evaluate the challenges encountered in the Urban Family Physician Program, with a particular focus on the perspectives of insurance organizations.

**Methods:**

A qualitative approach was adopted, involving semi-structured interviews with 22 experts and managers from basic health insurance funds. Snowball sampling facilitated participant selection, and interviews proceeded until saturation. Data analysis utilized content analysis and Atlas-T software, adhering to COREQ criteria.

**Results:**

Implementation problems of the urban family physician program were categorized into ten Categories and 22 Subcategories, including financing, stewardship, human resources, structure, culture, information system, payment, monitoring and control, the function of insurance organizations, and implementation.

**Conclusion:**

The urban family physician program’s implementation challenges, as viewed by health insurance organizations, underscore the necessity for strategic decision-making in financing, payment models, electronic system integration, structural adjustments, comprehensive monitoring, evaluation, cultural considerations, and appropriate devolution to insurance entities.

**Supplementary Information:**

The online version contains supplementary material available at 10.1186/s12889-024-19434-5.

## Background

The pursuit of ‘health for all’ is a global imperative, underscored by the commitment of the World Health Organization (WHO) in 1978. This vision, an integral part of the sustainable development agenda, places primary health care (PHC) and universal health coverage at the forefront of international health policy [1, 2].

The family physician (FP) program is among the endeavors to achieve this goal [3].Countries such as the United Kingdom, Denmark, Norway, Spain, and the Netherlands have demonstrated the efficacy of the FP program as a cornerstone of their health systems [4]. The Iranian healthcare system initially implemented the FP program in 2004, following the fourth and fifth national development plans, which underscored the importance of establishing a health insurance system through the FP and referral system. Furthermore, the 2014 overarching health policies also indicated the implementation of this program. In accordance with the National Annual Budget of 2005, the medical insurance organization was obliged to provide healthcare insurance to individuals residing in rural areas and cities with a population of less than 20,000 through the FP and referral system program [5]. Subsequently, the urban FP program was piloted in two provinces, Fars and Mazandaran, in 2012 [6].

A series of studies have demonstrated that FPs are capable of providing more effective preventive and curative services, while simultaneously utilizing fewer resources. Furthermore, they have been shown to possess the capacity to more effectively control the spread of certain diseases [7]. It is possible to meet the needs of nearly 80 to 90% of the population at the first level of service provision. A study of 29 European countries found that individuals with chronic diseases had a better status in countries that benefited from robust and cohesive primary healthcare (PHC) services [8]. Conversely, inadequate attention to FP and the referral system can disrupt the continuum of healthcare delivery, resulting in increased costs and a decline in quality [9].

The successful implementation of FP is contingent upon a multitude of factors, including sustainable financing, strict payment rules, training of personnel and staff, media contribution, revising the medical education curriculum, engaging the private sector as well as professionals, ensuring the continuation of the program, designing an appropriate structure and developing protocols, intra- and inter-sectoral collaboration, and advocating for the support of policy-makers [10].While the program has achieved achievements, including an increased rate of disease detection, declined out-of-pocket expenses, higher access to healthcare services, and promoting community health [11], it also suffers from limitations [12]. Mehraulhasani et al. identified key challenges in the urban family physician program, especially the interaction between the health system and insurance [13]. Lankarani et al. identified a number of challenges in the field of insurance, including limited resources, inefficient interaction between the ministries of welfare and health, the existence of multiple insurance organizations, defective service purchase mechanisms, and delays in payment to family physician [14]. Some experts in the study acknowledged that the benefits package is not aligned with the time frame and population covered by physicians [15].

To understand the multifaceted challenges of implementing this program, it is essential to consider the perspectives of various stakeholders, particularly those of insurance organizations. These entities play a pivotal role in policy-making and the practical execution of health programs. Their insights are invaluable for identifying financial, administrative, and systemic barriers that may impede the program’s success. Moreover, insurance organizations’ involvement is crucial for ensuring the sustainability, quality, and continuity of care provided under the family physician program. By incorporating the views of these key stakeholders,, this study intended to identify challenges of FP and referral system program following the perspective of basic health insurance organizations. Although numerous studies have been conducted in this field, no specific study has been carried out from the perspective of insurance organisations (the most significant stakeholders in the programme), which is the focus of this study. The objective is to identify and prioritise implementation solutions to address the challenges of the programme. Therefore, the objective of this study is to identify and prioritise implementation solutions to address the challenges of the Family Physician programme in Iran.

In order to gain a full understanding of the complex challenges involved in implementing this program, it would be beneficial to consider the perspectives of a range of stakeholders, with particular attention paid to those of insurance organizations. These entities play a significant role in policy-making and the practical execution of health programs. Their insights are invaluable for identifying potential financial, administrative, and systemic barriers that may impede the program’s success. It would be remiss of us not to mention the crucial role that insurance organizations play in ensuring the sustainability, quality, and continuity of care provided under the family physician program. It was our intention in this study to identify the challenges of the FP and referral system program from the perspective of basic health insurance organizations by incorporating their views. While there have been many studies conducted in this field, there has been a notable absence of studies carried out from the perspective of insurance organizations (the most significant stakeholders in the program), which is the focus of this study. The objective is to identify and prioritize implementation solutions to address the challenges of the program. Therefore, the objective of this study is to identify and prioritize implementation solutions to address the challenges of the Family Physician program in Iran.

## Methods

### Study context and sampling strategy

The present study focused on the urban family physician program in Iran, with a particular focus on the provinces of Mazandaran and Fars. The urban family physician project in Iran is currently being implemented in the provinces of Mazandaran and Fars. Additionally, managers, experts, and policy-makers related to the urban family physician program and basic insurance are also based in Tehran. In this study, we attempted to utilize all three provinces for interviews, with the interviews continuing until data saturation was reached. The initial participants were selected purposefully based on the knowledge of the researchers and the lack of knowledge of other experts in the field. The snowball method was then employed to select further participants in the study. This approach ensured a diverse and comprehensive representation of perspectives, which was crucial for the depth of our qualitative analysis.

The evidence indicates that health insurance organizations perceive the urban FP program to be constrained and fragmented. Consequently, a content analysis was conducted using the Elo and King approach, which comprises three stages: preparation, organization, and reporting [16]. The complexity of the meaning, the holistic perspective, and the contextual dependence of the content are among the reasons why content analysis can be utilized as a qualitative research method. Given the nature of this study, which requires the analysis of this issue and the links between them, as well as the revelation of the hidden links between them, this method was deemed appropriate.

### Data collection

Semi-structured interviews were conducted from August 2021 to December, with questions crafted to prompt detailed discussions on the challenges of the family physician program. The interview guide was developed following an extensive literature review and consultations with subject matter experts, including the research supervisor and advisor. Interviews were conducted until data saturation was reached, after 22 interviews, each lasting approximately 60 min. To ensure accuracy, all interviews were audio-recorded and transcribed verbatim, complemented by meticulous field notes. The majority of the interviews were conducted in the interviewees’ offices.

### Data analysis

We adopted Elo and King’s content analysis approach, which allowed us to systematically categorise the data. Our inductive analysis was rigorous, with continuous immersion in the data to ensure a holistic understanding.

To maintain the scientific accuracy and trustworthiness of our qualitative data, we implemented a four-criteria assessment [17, 18]: trustworthiness, transferability, dependability and fidelity. Trustworthiness was enhanced by member checking, where respondents validated the interviewer’s understanding. Transferability was demonstrated through the purposive selection of a diverse range of managers and experts in the central office (Tehran) and two provinces. Reliability was achieved through detailed documentation of the research process and a pre-established protocol. Conformability was maintained by involving the research team in in-depth discussions and by having participants review the findings to mitigate bias.

Reflexivity is a tool that makes qualitative studies more credible. Researchers should be aware of the influence of their previous life experiences as in previous studies and should look back to assess their effect [19] In this study, the researchers wrote their reflections and worked on them by interviewing experts and codifying and analysing the data. While this idea was discussed with the research group, The initial findings were presented in a meeting in the presence of professors. Besides, the edited text of the interviews has been sent to the participants for confirmation.

Also, the research purpose and methodology were subjected to scrutiny by the Internal Research Ethics Committee of the university (IR.SBMU.SME.REC.1400.025). The study procedure, as well as its goals, were explained to the participants, and they signed the written consent before entering the study. In addition, they were ensured of the confidentiality of the data. The combined criteria were used to report qualitative studies Consolidated Criteria for Reporting Qualitative Research (COREQ), which includes 32 items (Additional file 1) [20].

## Results

A total of 22 in-depth interviews were performed Table 1. The findings were categorized into ten categories and 22 Sub-categories Table 2.

**Table 1:** Participant’ demographic features

Participant	Age	Education level		Work experience	Work field				Position	
		Bachelor	Ph.D			Insurance organization - fars	Insurance organization - province	Insurance organization - Tehran	Expert	Manager
P1	30–40		*	> 10				*	*	
P2	> 50		*	> 20				*		*
P3	> 50		*	> 20				*	*	
P4	> 50		*	> 20				*		*
P5	> 50		*	> 20				*		*
P6	> 50		*	> 20				*	*	
P7	> 50		*	> 20				*		*
P8	> 50		*	> 20			*		*	
P9	> 50		*	> 20			*			*
P10	> 50		*	> 20			*			*
P11	40–50		*	> 20			*		*	
P12	> 50		*	> 20			*		*	
P13	> 50	*		> 20			*		*	
P14	40–50	*		> 20		*			*	
P15	> 50	*		> 20		*			*	
P16	> 50		*	> 20		*				*
P17	40–50		*	> 20		*			*	
P18	> 50		*	> 20		*				*
P19	40–50		*	> 20		*				*
P20	40–50	*		> 20			*		*	
P21	30–40		*	< 10				*	*	
P22	> 50		*	20				*	*	

**Table 2:** Challenges of the urban family physician program

Categories	Subcategories	Categories	Subcategories
**Financing**	▪ Lack of appropriate financing mechanism ▪ The high cost of implementing the program.	**Payment mechanism**	▪ Lack of balance between capitation rate and inflation rate ▪ Simultaneous payment of capitation and fee-for-service ▪ Delayed payment ▪ Inappropriate determination of the capitation rate
**Stewardship**	▪ Inappropriate policymaking ▪ Inappropriate inter-sectoral leadership ▪ Inadequate governance within the program.		
		**Monitoring and control**	▪ Insufficient evidence for program evaluation ▪ Inadequate program monitoring
**Human resources**	▪ Insufficiency of human resources ▪ A community-oriented approach is lacking within medical education.		
		**Function of health insurance organizations**	▪ The unrealistic role of insurers
**Structure**	▪ Lack of family physician structure	**Implementation of program**	▪ Program Objectives Lack Discernible SMART Criteria ▪ The service package is excessive yet superficial. ▪ Challenges in Program Implementation ▪ Challenges in Family Physician Roles
**Culture**	▪ Lack of a widespread culture of using services provided by family physicians		
**Information system**	▪ Inadequate generation and utilization of evidenc ▪ Lack of decision support systems		

### Financing

Funding refers to the mechanism by which financial resources are allocated for the implementation of the family physician program. The three main goals of a financing system are collection, pooling, and management of resources, as well as the purchasing of healthcare services. [21]. The participants identified two subcategories: **the lack of an appropriate financing mechanism** and **the cost of implementing the program**. 14 Interviewees mentioned the lack of an appropriate financing mechanism. A number of interviewees indicated that the financial resources for this programme were not considered, or if they were considered, they were not allocated to the insurance companies or the Ministry of Health did not provide them to the insurance companies. Conversely, some respondents also identified the high cost of the programme as a potential barrier to its implementation.A manager noted:

*The Ministry of Health and Medical Education is the main steward of the program. However*,* health insurance organizations reimburse the services*,* and there is no particular line of budget for the family physician program*,* and insurance organizations use their internal resources to pay the costs* (P15).

This issue inherently results in financing instability. 8 managers notedthat:

*‘There are earmarked resources for the rural family physician program. Nevertheless*,* there are no such funds for the urban program*,* as it is a pilot program*,* leading to limitations and resistance from people and providers. Therefore*,* there are no resources*,* and it may continue in the future. For instance*,* health insurance organizations were obliged to reimburse physicians and 30% of pharmaceutical costs*,* and the MoHME was supposed to pay the rest. Nevertheless*,* the MoHME did not pay any money from resources allocated to the program in the 2012 annual budget* (P1).*’*

Although the fifth national development plan emphasized the aggregation of health insurance funds, no significant measure has been implemented yet. An interviewee noted:

Health insurance funds were supposed to be aggregated, but, in reality, the dispersion is increasing, leading to lower pooling (P1).

The high cost of the program was among the issues noted by interviewees. Obviously, implementation of the program requires more resources in the first years, but over time it will lead to lower costs. An interviewee noted:

It is expected that implementation of the family physician program results in declined costs, with high investment return. For instance, UK and Italy reported that the investment return is achieved after 20 or 30 years (P15).

### Stewardship

The MoHME is the main steward of the health system. In other words, the MoHME must have overall supervision, guide the system, and develop and implement national measures on behalf of the government [22]. The interviewees identified the issue of inappropriate policy-making as a significantchallenge. The lack of political will, the existence of parallel programmes, the problems of the macro-management system, the inappropriate policymaking, the discontinuation of policies with changes in governments, the lobbying, the lack of a systemic approach, the conflict of interest, the fear of reforms, and the lack of domestication are all factors that contribute to improper policy-making. As one of the interviewees stated:

Lack of political will at the macro level to implement the program is the main challenge (P3).

*The Social Security Organization developed the Family Physician Instruction in 2008. Then*,* it was revised in 2010*,* and the program was piloted in Gilan province*,* named**** Trusted Physician****(Pezeshk Amin* (P4).

*The overall management system of the country affects the health system. Hence*,* if it suffers from significant challenges*,* the health system will also suffer (P5).*

When the new government came to office and began the health transformation plan, the family physician was taken away from the agenda. While almost a year ago, there was much emphasis on implementing the family physician program (P3).

I wish governments initiate only one good program when they come to the office, not the end of the government because it is well proved that new governments show no commitment to continue previous programs (P7).

*‘I think our health system suffers from one major problem and the others root in this problem*,* and the problem is the conflict of interest. Hence*,* if we could address conflict of interest*,* the political resistance would disappear*,* tariffs would be realistic*,* etc. (P3).’*

*For all ministers*,* continuing the status quo is much easier (P5).*

We should pay more attention to domestication. Why do we need a family physician? Why should we not use a health promoter program? The number of family physicians is significantly lower than other providers, and the program is more costly than other programs (P4).

Several interviewees emphasized the necessity for more effective **intersectoral leadership**. Intersectoral leadership refers to the strategies used by health policymakers to engage and persuade other sectors to work towards objectives that are consistent with enhancing public health. They declerd that related institutions do not adequately participate in the design and implementation of the program. Additionally, the roles of stakeholders have not been clearly delineated, and there is a paucity of interaction among them. One of the interviewees asserted that:

It seems that none of the effective components in health have been used for the family physician. The governor’s office, the radio station, the governor’s office, the policy makers have not come. It would be good to know what their role is. They should be at different stages to solve the problems related to the programme(P12).

One of the managers declare that:

*‘tasks are not clearly defined*,* and the individual responsible for overseeing the programme is not specified. For instance*,* it is unclear who should resolve the discrepancy between urban and rural family doctor programmes(P10)’.*

Some interviewees indicated that there is a lack of interaction between multiple stakeholders. Three interviewees declared that the family physician programme lacks a supervisor, and that the roles of stakeholders should be more carefully considered when designing the programme. One manager stated that:

*‘Some personnel at the Tehran headquarters prepared version 03 of the family doctor programme without adequately considering operational issues. It is imperative that there is a consensus to reduce the potential for harm (P14)’.*

Another identified challenge is the lack of proper governance within the sector. In the context of health systems, governance refers to the utilization of all available mechanisms to align operational activities within the sector with the policies of the sector. The interviewees acknowledged the existence of weak laws in this field. The program’s affiliated institutions were perceived as lacking essential participation for effective implementation. Furthermore, the role of beneficiaries remained ambiguous, and there was insufficient interaction among them. As they stated, *‘at the moment*,* the insurance companies operate according to their own discretion*,* and since they do not have a specific legal duty in this field*,* they can not continue if they do not want to. (P10) ’*.

### Human resources

The human resources of a health system represent its most valuable asset, as they are essential for ensuring the health and social justice of the population [23]. The participants identified two subcategories within this theme. The first subcategory, insufficiency of human resources, encompasses **the lack of sufficient personnel to meet the demands of the health system**. The second subcategory, **medical education not community-based**, addresses the discrepancy between the principles of community-based programs and the quality of training family physicians.

Another manager noted that: *‘the physician per population is also important and the country is faced with a shortage of physicians (P7).’* An interviewee declared that: *‘family physicians are not well-trained to act as a family physician (P12).’*

### Structure

In accordance with Paragraph A of Article 137 of the Fourth Development Plan obliged the government to redesign the structure of executive agencies and ministries in accordance with the policies and rules of this law and the experience of other countries [24]. he participants observed that there is no suitable structure for the family physician program.

We are faced with a shortage of resources and organizations; at least there should be a structure specially devoted to this program at the deputy for public health of the MoHME. The same is true for headquarters of health insurance organizations; there should be an office named Family Physician (P10).

### Culture

The high number of cultural determinants of the health system, on the one hand, and ignoring expert opinions regarding health culture and nonadherence to efficient and effective cultural patterns in the health system, on the other hand, resulted in the slowness of the health system [25]. The participants identified a subcategory: **the lack of a widespread culture of using services provided by family physicians**. In other words, the participants indicated that one of the challenges associated with the implementation of the family physician program is that it is primarily focused on the expertise of the medical professionals involved, which has led to a lack of trust in general physicians. 6 participants mentioned the challenges in the field of culture .A manager noted, *‘All people prefer to refer to specialists without clear reason. Therefore*,* it is far from expected to trust a GP who considers all aspects of their health (P12)*.’ Another manager noted, *‘the referral system had limitations*,* and cultural aspects of the program were not considered; hence*,* people preferred to use other mechanisms (P6).’*

### Information system

Reliable and timely health information is the foundation of public health activities. Consequently, without robust data collection, analysis, dissemination, and administration systems, decision-makers are unable to identify problems and needs, track improvements, assess the impact of interventions, and make evidence-based decisions [26]. The participants identified two sub-categories: generation and application of evidence and decision support systems. Eleven interviewees highlighted challenges regarding the generation and administration of evidence in information systems. A manager noted that: *‘Currently*,* the SIB system is available*,* but it does not include reports. Or*,* regarding the electronic prescription*,* it contains no link for electronic prescription while it is mentioned in the 02 version of its constitution (P15).’* Another manager noted: ‘We only collect data, with no analysis or report (P15).’

6 participants noted: *‘physicians enter the date to the information systems by the MoHME. But these systems did not send data to us*,* and it’s about a week since this connection was established (P5).’* another person noted: *‘There are different and various information systems among provinces and even in cities*,* leading to difficulties in aggregation and analysis of data (P9).’*

### Payment

The participants identified several challenges, including an **imbalance between the growth of the per capita rate and the inflation rate**, t**he simultaneous payment of the per capita rate and the fee-for-service**, **delayed payment**, and **the lack of evidence to determine the per capita rate**. One participant observed that the participants held the view that the per capita rate was not proportional to the inflation rate., *‘it was decided to pay 12% of the per capita rate of physicians to healthcare staff in 2012. However*,* this amount does not equal the minimum wage (P14). A number of interviewees highlighted the irregularities in the remuneration of physician.’This year per capita rate was increased by 50% Meanwhile*,* physicians receive both premium and FFS*, i.e.,* they are mixed up with each other (P4)’*. Some participants expressed the view that remuneration to family physicians and their teams is frequently delayed, which has a detrimental effect on the motivation of the family physician team. One participant stated that *“The doctor is informed that their financial relationship with the patient is broken and that they are now a salaried employee. The individual who previously received perks from the family physician team has now become their salary. If the family physician does not receive payment at the beginning of the month*,* they will have to resort to other means of financing. However*,* this is now a less problematic situation*,* with payments being made on time(P12)”.* Some of the interviewees expressed the opinion that the per capita determination lacked scientific basis. *‘The other fact is not using evidence to determine the per capita rate. We should be aware of whether there is a balance between provided services and costs (P18).’*

### Monitoring and control

An effective monitoring system is crucial to achieving program goals [27]. Four participants noted challenges in the lack of evidence for program evaluation, while emphasizing two subcategories: the absence of cost-effectiveness studies at the provincial level and the failure to investigate the consequences of making referral optional. Some interviews indicated that the transition from a mandatory to an optional referral system may have introduced new challenges to the program. Some have expressed the view that the program’s effectiveness studies and their evaluation have not been conducted in an optimal manner. One participant noted that: A participant noted: *‘there is no investigation on our status before and after implementing the program or to compare pilot provinces with other provinces (P14)’. ‘They were supposed to do this in four months and to perform an investigation to compare the costs. However*,* this investigation is not performed still (P16).’* 11 participants also mentioned challenges related to **monitoring** with two sub-categories of lack of a systematic monitoring program for providers and lack of supervision of high-level institutions on operational units: *‘Our regulatory mechanisms are not activated. The monitoring infrastructure is not available. There’s a checklist*,* but it doesn’t provide the right basis for evaluating the performance of physicians (P4).’*

The relationship between insures and health insurance organizations is weak. There is no mechanism to engage insures in monitoring, as they are the main stakeholder. When such a relation was established, it was weak (P12).

High-level authorities should monitor other stakeholders (e.g., the parliament, judiciary system, and planning and budget organization), but they do not supervise (P2).

### Functions of insurance organizations

Seven participants identified the **unrealistic role of health insurance organizations** and the necessity for sufficient authority. In essence, the insurance companies were deemed to lack the requisite authority to oversee the implementation of the urban family physician programme. The role of these organizations was perceived to be primarily financial, with no involvement in decision-making at either the micro or macro level. Moreover, they were not consulted for advice. A manager noted: *‘We have no insurance mechanism (an organization that follows the concept of insurance and acts as an agent (P2).’* Another manager noted, *‘Insurance organization notices that a laboratory does not use a particular kit*,* but claims contain all items. Nevertheless*,* they do not have the authority to address this problem (P12).’*

### Implementation

Three participants identified challenges in **setting goals**. The program’s objectives were not subject to any discernible criteria, such as compliance with the SMART model. One interviewee noted that *‘the start and finish points are not clear. I mean policymaking is not performed*,* and the implementation method is also unclear (P9).’* One participant highlighted the difficulties associated with the benefit package. Some participants identified the service package as being too extensive, while others perceived it as lacking in depth. A manager noted: *‘the benefit package is extensive*,* but its depth is small and financial protection is not good (P7).’* Fifteen participants identified implementation challenges. *‘the pilot program took a long time*,* and the lack of national implementation has caused the program to be marginalized in two provinces*,* causing a dichotomy (P6)*.’ The participants identified a number of challenges in the implementation process. The team identified a number of issues in the implementation process, including the lack of clarity regarding the programme tasks, the multiplicity of implementation versions, the differences in programme implementation between the two provinces, the discrepancy between the compiled programme and its implementation, and the optional referral system. Three participants observed that there are multiple models for implementing the FP. *‘Now we have several models of the FP in the country. Rural and urban family physicians in Mazandaran and Fars and referral programs in ten provinces*,* which are different from each other. (P7).’* Another problem roots in the fact that referral is optional. A manager said: *‘the last shot to the FP was making referral optional (i.e.*,* in Fars province)*,* which made a program with no significant effect (P7).’* Seven participants identified challenges associated with family physician roles. The participants identified a number of issues, including deficiencies in the family physician’s gatekeeping role and a lack of comprehensive care. In other words, the participants believed that many clients sought the family physician in order to benefit from the referral. By affixing their signature to the referral sheet, the family physician could then refer the client to the second or third level of referral. Conversely, they held the view that the family physician’s role in the city was passive, with a tendency to neglect active follow-up of the population under their care. Their focus was on treatment rather than prevention. *‘Many people refer to FPs to receive a referral note to a specialist with a lower premium. On the other hand*,* the information exchange is not enough because the communication infrastructure between physicians and patients is not provided. Referral forms of patients are often lost in a specialist’s office and are not referred to the family physician (P16).’*

3 participants mentioned premiums as another problem. *‘The high council for health insurance determines the premium*,* and the fact that the referral is optional has turned the program against itself (P1).’* Participants emphasized ‘*that FP is mostly a curative program. For instance*,* in Fars province*,* the FP is mostly curative than preventive (P19).’*

## Discussion

The evaluation of the urban family physician (FP) program, which entails determining the level of achievements, paves the way to identify field challenges and plan to address them. This, in turn, facilitates the national-wide implementation of the program. The findings help to identify field problems of the urban FP program in Iran. The majority of participants believed that the insufficient pool of resources was a consequence of the high number of health insurance funds, the lack of financial resources allocated to health insurance organizations, and the unsustainability of resources. This has led to difficulties in the allocation of resources and the inability to expand the program nationally. Article 10 of the overall health policies emphasized the importance of sustainable financing of healthcare resources [28]. Mehtarpour et al. [29] also emphasized the importance of sustainable financing for healthcare services. Akbari et al. [30] also identified financing as a significant factor influencing the accumulation of insurance funds.The participants in this study believed that lack of will and commitment of senior officials to law enforcement and its effects on the stagnant state of programs, lack of a systematic approach among managers, negative influences of organizations and influential individuals who are not aware of health system reforms and its mechanisms, lack of domestication of programs and reforms despite differences in the social, economic, political, and cultural context of countries, change of governments and disinterest in continuation of programs initiated by previous governments, not engaging stakeholders, and weakness of and nonadherence to laws pave the way for the vulnerability of programs. In a study that employed the perspectivesof policymakers, executive managers, and experts of the FP program at the MoHME, health insurance organizations, and the parliament, Shiani et al. [31] mentioned the challenge of the substantiveness of programs and the effect of managerial changes. Mohammadi Bolbanabad et al. identified challenges related to the stewardship of the rural FP program [32]. Given the involvement of numerous stakeholders in national-level programs, such as FPs, it is evident that fostering an interactive context is of paramount importance to achieve objectives. Otherwise, the dispersion and disharmony of stakeholders result in the waste of resources and failure to achieve the goals.

A number of participants highlighted the lack of sufficient human resources in terms of both quantity and quality. In particular, they noted the insufficient number of human resources and the failure to adopt a community-based approach. In addition to a lack of human resources, a lack of attention to motivational factors and a weakness in community-based education were identified as contributing to a decline in the quality of the services provided. In a study that investigated the perspective of managers, Kabir et al. [33] noted the high number of people living in catchment areas of physicians. Behzadifar et al. [34] identified motivational factors as a challenge in the implementation of a rural family planning program. It appears that even if a sufficient number of family planning (FP) services are available, the absence of gatekeeping skills and a community-based approach may result in suboptimal outcomes. Kabir et al. emphasized the significance of equipping health teams with the requisite skills to fulfill their duties in accordance with the fundamental benefit package [35]. One participant highlighted the challenge of establishing the necessary structure for implementing the program within health insurance organizations and medical universities. The participants contended that these organizations should have an appropriate structure based on the defined goals to enhance accountability. Other studies did not report such findings. As the Deputy of Public Health of MoHME is responsible for the program’s leadership, and its implementation should be performed in collaboration with the Deputy for Treatment Affairs of the MoHME, there should be a specialized structure at these deputies. A significant number of participants expressed the view that cultural challenges were rooted in the lack of social marketing and medicine specialisation. It was observed that social marketing plays a significant role in influencing attitudes and acceptance of healthcare reforms among the general public. One of the cultural barriers to implementing the FP program is the public’s trust in specialized doctors, rather than GPs. The implementation of preventive interventions and referral chains using the FP program is contingent upon the community’s support and participation. As observed by GolAlizadeh et al. [36], several studies have highlighted issues pertaining to the cultural aspects of the programs in question. Alaee et al. [37] also observed that the successful implementation of programs necessitates the simultaneous investment in health systems and people. Consequently, studies have recommended the promotion of services through the referral system and the strengthening of FP through the use of media, localized methods, and long-term plans that intend to establish the referral system through schools.The following issues have been identified as problematic in the context of the health information dimension: data inaccuracy, data poverty, lack of access to data, weakness of and diversity of systems, and managers’ unawareness about data analysis. In a study conducted in Kerman, Mehralhasani et al. [38] identified the inefficient management of health information as one of the seven challenges facing the rural FP program.The efficacy and quality of healthcare services are contingent upon the functionality of health information systems and technologies. Any deficiencies or disruptions in the role and function of these systems result in a decline in efficiency and quality of care, as well as an interruption in the decision-making process. Additionally, the manner in which healthcare services are compensated affects healthcare expenditures, out-of-pocket expenditures, and the quality of services. Therefore, a simple decision for payers would be to alter the payment method or utilize a combination of methods. A number of studies, including Abedi et al. [39], have highlighted the issue of payment methods. It appears that the exact determination of the per capita rate based on the inflation rate would result in a more favourable living standard for physicians and other healthcare staff, as the first line of service provision. Furthermore, it would lead to increased staff motivation, which would have a positive impact. For instance, Schweizer noted the payment mechanism as a motivational factor for staff [40]. All organizations require monitoring to ensure their continued survival and to ascertain the quality of their performance and the implementation of their programs. Examples of monitoring include investigating the status of the organization before and after implementing a program, comparing pilot and non-pilot areas, and studying the effect of interventions. In order to make decisions regarding the expansion and implementation of programs and the exit of pilot programs, evidence is required. Such studies provide a basis for evidence-based policymaking. Some interviewees noted that the current checklists for monitoring and evaluation are not appropriate or effective. They believed that although continuous monitoring and evaluation are crucial for healthcare organizations, developing a comprehensive tool, model, or indicator to measure all aspects affecting the quantity and quality of performance and identifying the strengths and areas that require quality improvement is a prerequisite of this process.

In accordance with Article 91 of the Fourth National Development Plan, health insurance organizations are obliged to implement the requisite mechanisms for family physician-based health insurance schemes. It appears that failure of health insurance organizations to achieve strategic purchasing will result in challenges to the program and the inability to achieve the defined goals. The majority of respondents emphasized the necessity of a certain level of authority to perform this role.

It was observed by participants that the level of authority of insurance organizations, particularly with regard to monitoring and control, pricing, and benefit packages, is constrained, which gives rise to difficulties in the fulfillment of the primary responsibilities of these organizations. With regard to the FP program, the pivotal role of health insurance organizations is that of purchasing services, rather than merely financing resources without intervention or providing operational strategies. Insurance organizations expect to be able to apply their perspectives as stewards of insureds, with sufficient authority to determine strategies and evaluate the performance of the FP program. Shirjang emphasized the necessity of supervision of service purchasers (i.e., health insurance organizations) and the importance of having sufficient legal authorities [41]. It appears that, as all programs have goals, any deficiency in setting goals, including timing, not setting goals, not determining implementation method, and inability to achieve goals, will have a detrimental impact on the quality of subsequent stages. It was perceived by participants that the prolonged piloting of the program had resulted in policymakers becoming indifferent to the need to make a final decision and address the issues being experienced by the pilot provinces [42]. Furthermore, participants highlighted the multitude of implemented programs. Some managers contended that the pace of change in the program, coupled with the emergence of new versions of the family physician program, has rendered the system incapable of administering new versions. It was observed that there were significant differences between the two provinces with regard to the implementation of the program. Consequently, it was deemed inappropriate to attempt to make managerial decisions based on comparisons between the two provinces. The differing rural and urban populations of the two provinces, the extent of the program, and the contracting mechanisms (e.g., with physicians in Mazandaran province and clinics in Fars provinces) were identified by participants as factors that require consideration. Participants contended that the option of making referrals had a detrimental impact on the program. Deficiencies in the gatekeeping role of FP, financial relationships between physicians and patients, and a failure to adopt a community-based perspective indicate problems such as the passive role of FPs, a curative approach (as opposed to a health-oriented one), and the receipt of premiums.

## Conclusion

The findings indicated potential areas for improvement in the implementation of the urban FP program in Iran, based on the perspective of health insurance organizations. In terms of financing, there may be opportunities to explore the aggregation of health insurance funds and the earmarking of resources for implementing the program. Health insurance organizations could also consider ways to play their real role as strategic purchasers, which may involve delegation of authorities from the ministry of health to insurance organizations, as well as advocacy. It is also suggested that the Ministry of Health and Medical Education (MoHME) consider revising the medical curriculum and providing in-service training for FPs, developing appropriate electronic infrastructure and integrating systems, and creating a structure tailored to the FPs in medical universities and insurance organizations.

Other recommendations include considering the socioeconomic status of the country when formulating and implementing a single version and appropriate operational plan, and creating an appropriate cultural context. It would also be beneficial to establish a mandatory referral system by the MoHME, the combined supervisory role of the MoHME and health insurance organizations, and provide appropriate evaluation tools beyond the checklists and traditional methods. Additionally, it would be advantageous to encourage the participation and cooperation of organizations related to the program through parliament and the judiciary. It would be beneficial to conduct further research in order to address the significant evidence gaps concerning four key areas: (1) evaluating the impact of different program versions, (2) exploring the feasibility of separating payments to health care providers from family doctors, (3) determining an equitable per capita rate for individuals covered by family doctors, and (4) identifying appropriate evaluation tools. By addressing these gaps, we can hope to optimize family doctor programs and improve overall health outcomes.

### Electronic supplementary material

Below is the link to the electronic supplementary material.

Supplementary Material 1

## Data Availability

No datasets were generated or analysed during the current study.

## Abbreviations

FP: Family Physician
WHO: World Health Organization
PHC: Primary Health Care
MoHME: Ministry of Health and Medical Education
