# Supplementary material for: Assessing urban family physician program challenges in Iran: the insurance organizations’ perspective(2021)
Source: BMC Public Health. 2024 Jul 20;24:1947. doi: 10.1186/s12889-024-19434-5 (PMC11265013; doi:10.1186/s12889-024-19434-5)
Supplement: Supplementary file 1 — Supplementary Material 1 [file 12889_2024_19434_MOESM1_ESM.docx]

**Consolidated Criteria for Reporting Qualitative Research (COREQ) 32-item checklist - Assessing Urban Family Physician Program Challenges in Iran**

| **No Item** | **Guide question/description** | **Answer** |
| --- | --- | --- |
| Domain 1: Research team and reflexivity | | |
| **Personal Characteristics** | | |
| 1 Interviewers/ facilitators | Which author/s conducted the interview or focus group? | It was noted in the methodology section that the correspoiunding author (FM) |
| 2 Credentials | What were the researcher's credentials? | In the research paper, the research team's qualifications and affiliations were listed. |
| 3 Occupation | What was their occupation at the time of the study? | The research team's occupations were given in the affiliations section |
| 4 Gender | Was the researcher male or female? | The methods section noted that the analysis team comprised of two female academics |
| 5 Experience and training | What experience or training did the researcher have? | The analysis was undertaken by a research team with rich experience performing qualitative research in healthcare settings, as stated in the methods section |
| **Relationship with participants** | | |
| 6 Relationship established | Was a relationship established prior to study commencement? | This was not stated in the study. Before the study, none of the research team had relationships with any subjects. |
| 7 Participant  knowledge of the  interviewer | What did the participants know about the researcher? For example, personal goals and reasons for doing the research | The interviewer called people to arrange the interview and then sent the interview guide via email or social networks. The interview was then conducted face-to-face, and the purpose of the interview was explained at the beginning of the meeting. |
| 8 Interviewer characteristics | What characteristics were reported about the interviewer/facilitator? For example, bias, assumptions, reasons, and interests in the research topic | The interviewees were selected from people who have experience working in the insurance organization of Fars, Mazandaran and Tehran provinces. The interviewees should have the details of the family physician implementation program in the cities. |
| **Domain 2: Study design** | | |
| **Theoretical framework** | | |
| 9 Methodological orientation  and theory | What methodological orientation was stated to underpin the study? For example, grounded theory, discourse analysis, ethnography, phenomenology and content analysis | content analysis (methods, paragraph 2) |
| Participant selection | | |
| 10 Sampling | How were participants selected? For example, purposive, convenience, consecutive, and snowball | Purposeful sampling method (methods, paragraph 1) |
| 11 Method of approach | How were participants approached? For example, face‐to‐face, telephone, mail, and email | Face-to-face interviews have been conducted with the participants |
| 12 Sample size | How many participants were in the study? | 22 (methods, paragraph 1) |
| 13 Non‐participation | How many people refused to participate or dropped out? Reasons? | All the selected people agreed to participate in the study after explaining the objectives of the study |
| **Setting** | | |
| 14 Setting of data collection | Where was the data collected? For example,home, clinic, and workplace | The interview was conducted at the workplace |
| 15 Presence of non‐participants | Was anyone else present besides the participants and researchers? | NO |
| 16 Description of sample | What are the important characteristics of the sample? For example, demographic data and date | The selection criteria for the study included job titles and service records of the people participating in the study. (Table1) |
| **Data collection** | | |
| 17 Interview guide | Were questions, prompts, guides provided by the authors? Was it pilot tested? | Yes |
| 18 Repeat interviews | Were repeat interviews carried out? If yes, how many? | There were no follow-up interviews, as noted in the methods section. |
| 19 Audio/visual recording | Did the research use audio or visual recording to collect the data? | Yes, Audio recording |
| 20 Field notes | Were field notes made during and/or after the interview or focus group? | Field notes were used during the interview. |
| 21 Duration | What was the duration of the interviews or focus group? | The average duration of the interviews was 60 minutes. |
| 22 Data saturation | Was data saturation discussed? | The sample size was determined by the saturation of data, a process that was documented in the Methods section. |
| 23 Transcripts returned | Were transcripts returned to participants for comment and/or correction? | At the end of the interview session, summary of the written responses was provided to the interviewee. Subsequently, the transcript of the interview was sent to some of the interviewees via email. |
| **Domain 3: Analysis and findings** | | |
| **Data** **analysis** | | |
| 24 Number of data  coders | How many data coders coded the data? | Two authors (FM and LSH) performed data coding. And those who disagreed reached a consensus after discussion, and finally all the authors fully discussed and approved the coding framework. |
| 25 Description of the  coding tree | Did authors provide a description of the  coding tree? | The "Methods" section describes the code structure and conceptual map. |
| 26 Derivation of  themes | Were themes identified in advance or derived from the data? | Themes were derived inductive and deductive from data |
| 27 Software | What software, if applicable, was used to manage the data | Atlas-ti |
| 28 Participant checking | Did participants provide feedback on the findings? | The results of the study were sent to one of the insurance organizations and presented in the presence of some interviewees. |
| **Reporting** | | |
| 29 Quotations  presented | Were participant quotations presented to illustrate the themes/findings? Was each quotation identified? For example, participant number | Under the results section, participants' quotes were included. |
| 30 Data and findings  Consistent | Was there consistency between the data presented and the findings? | To verify interpretations and findings, the author and the supervisors evaluated and validated the final themes and subthemes. |
| 31 Clarity of major themes | Were major themes clearly presented in the findings? | The content analysis weighted all themes equally. |
| 32 Clarity of minor themes | Is there a description of diverse cases or discussion of minor themes? | Within the content analysis, all themes received equal weighting |
